# Alternative Dietary Patterns for Americans: Low-Carbohydrate Diets

**DOI:** 10.3390/nu13103299

**Published:** 2021-09-22

**Authors:** Jeff S. Volek, Stephen D. Phinney, Ronald M. Krauss, Richard J. Johnson, Laura R. Saslow, Barbara Gower, William S. Yancy, Janet C. King, Frederick M. Hecht, Nina Teicholz, Bruce R. Bistrian, Osama Hamdy

**Affiliations:** 1 Department of Human Sciences, Ohio State University, Columbus, OH 43017, USA; 2 Virta Health, San Francisco, CA 94015, USA; steve@virtahealth.com; 3 Departments of Pediatrics and Medicine, University of California, San Francisco, CA 94143, USA; ronald.krauss@ucsf.edu; 4 Division of Renal Diseases and Hypertension, University of Colorado Anschutz Medical Campus, Aurora, CO 80045, USA; richard.johnson@cuanschutz.edu; 5 Department of Behavior & Biological Sciences, University of Michigan, Ann Arbor, MI 48109, USA; saslowl@umich.edu; 6 Department of Nutrition Sciences, University of Alabama, Birmingham, AL 35233, USA; bgower@uab.edu; 7 Department of Medicine, Lifestyle and Weight Management Center, Duke University, Durham, NC 27705, USA; will.yancy@duke.edu; 8 Department of Nutritional Sciences & Toxicology, University of California, Berkley, CA 94720, USA; janet.king@ucsf.edu; 9 Osher Center for Integrative Medicine, University of California San Francisco, San Francisco, CA 94115, USA; rick.hecht@ucsf.edu; 10 The Nutrition Coalition, New York, NY 10011, USA; teicholz@gmail.com; 11 Beth Israel Deaconess Medical Center, Boston, MA 02215, USA; bbistria@bidmc.harvard.edu; 12 Joslin Diabetes Center, Harvard Medical School, Boston, MA 02215, USA; osama.hamdy@joslin.harvard.edu

**Keywords:** low-carbohydrate, diets, high-fat, insulin resistance, obesity, type-2 diabetes, dietary guidelines, eating patterns

## Abstract

The decades-long dietary experiment embodied in the Dietary Guidelines for Americans (DGA) focused on limiting fat, especially saturated fat, and higher carbohydrate intake has coincided with rapidly escalating epidemics of obesity and type 2 diabetes (T2D) that are contributing to the progression of cardiovascular disease (CVD) and other diet-related chronic diseases. Moreover, the lack of flexibility in the DGA as it pertains to low carbohydrate approaches does not align with the contemporary trend toward precision nutrition. We argue that personalizing the level of dietary carbohydrate should be a high priority based on evidence that Americans have a wide spectrum of metabolic variability in their tolerance to high carbohydrate loads. Obesity, metabolic syndrome, and T2D are conditions strongly associated with insulin resistance, a condition exacerbated by increased dietary carbohydrate and improved by restricting carbohydrate. Low-carbohydrate diets are grounded across the time-span of human evolution, have well-established biochemical principles, and are now supported by multiple clinical trials in humans that demonstrate consistent improvements in multiple established risk factors associated with insulin resistance and cardiovascular disease. The American Diabetes Association (ADA) recently recognized a low carbohydrate eating pattern as an effective approach for patients with diabetes. Despite this evidence base, low-carbohydrate diets are not reflected in the DGA. As the DGA Dietary Patterns have not been demonstrated to be universally effective in addressing the needs of many Americans and recognizing the lack of widely available treatments for obesity, metabolic syndrome, and T2D that are safe, effective, and sustainable, the argument for an alternative, low-carbohydrate Dietary Pattern is all the more compelling.

## 1. Introduction: The Current 2020 Dietary Guidelines Need Greater Flexibility

The current 2020-2025 Dietary Guidelines for Americans (DGA) recommends “Dietary Patterns” that provide little flexibility in the distribution of fat, protein, and carbohydrate. Relying on the “Acceptable Macronutrient Distribution Ranges (AMDR),” as defined by the National Academies, the 2020 DGA allows dietary fat to range from 20% to 35% of calories, and carbohydrate, from 45% to 65% [1]. Even more narrow ranges, modeled by the expert advisory committee for the DGA, show the government’s recommended Dietary Patterns to be 29–32% fat and 51–54% carbohydrate, as a percent of total energy [2]. It is unclear which standard will drive the federal government’s food and nutrition programs, but either can be considered relatively low in fat and high in carbohydrates, compared to the average American diet before the implementation of the DGA in 1980 [3]. In addition, the quality of carbohydrate consumed today is poor, with higher intakes of high-glycemic index carbohydrates including processed grains and simple sugars (e.g., high fructose corn syrup). These are narrow recommendations relative to the much broader range of carbohydrate consumed throughout human evolution [4], and the past or current DGA macronutrient recommendations clearly do not encompass a low-carbohydrate eating pattern. 

Despite the AMDRs, carbohydrate is not an essential dietary macronutrient as there is no minimum requirement that prevents deficiency symptoms [5]. Over half of Americans have a diet-related chronic disease with some degree of insulin resistance involving carbohydrate intolerance [6,7], and thus many could benefit from limiting carbohydrate intake. Because accurate quantification of nutrient intake in most human studies is lacking, we do not emphasize comparisons among different low-carbohydrate diets in this review. Our central point is that an alternative eating pattern, characterized by lower carbohydrate and higher fat intake than is recommended by the 2020 Dietary Guidelines, is supported by a substantial body of evidence. 

## 2. Low-Carbohydrate Diets Defined

There are no formal or universally accepted definitions for low-carbohydrate diets, although as the name implies, the key feature is a reduction in carbohydrate in the diet. Since variations in caloric intake significantly influence the percent of calories derived from carbohydrate at any given carbohydrate intake, it is preferred to define low-carbohydrate diets by their absolute content in grams. Currently, the U.S. Dietary Guidelines advise [8], and Americans typically consume, more than half of total calories derived from carbohydrate. Based on average caloric consumption data, this corresponds to a daily carbohydrate intake of more than 300 grams for men per day and 200 grams for women [9]. The National Academy of Sciences recommends a daily allowance (RDA) for carbohydrate of 130 grams. This carbohydrate requirement is presumably based on the minimum amount required to provide the brain with an adequate supply of glucose, although this rationale does not have a physiological basis, given that humans can make glucose from non-carbohydrate sources and that the brain can use alternative fuels like ketones. Nevertheless, these numbers provide context for determining a reasonable place to start in terms of defining low-carbohydrate diets. In alignment with others [10], we suggest that a definition of a low-carbohydrate diet is one consisting of fewer than 130 grams per day. This level of carbohydrate is a general threshold for purposes of broadly defining diets and does not necessarily reflect the wide variation in response to carbohydrate at the individual level. Because low-carbohydrate diets generally consist of no more than 130 grams per day (520 kcals) and moderate protein, the majority of other calories are derived from dietary fat. Thus, low-carbohydrate diets are often referred to as low-carbohydrate and high-fat (LCHF). 

Ketogenic diets (KD) are a subset of low-carbohydrate diets that usually consist of less than 50 grams carbohydrate per day with adequate but not excessive protein, and varying amounts of fat depending on the intended body weight goals. Energy content of KD can fluctuate from very low-calorie (e.g., semi-starvation, <800 kcal/day) to mildly hypocaloric to eucaloric diets [11]. Ketogenic diets aim to increase the production of ketones, in order to achieve a state of ‘nutritional ketosis.’ In nutritional ketosis, fatty acids and ketones rather than glucose become the body’s primary sources of fuel. In the keto-adapted state, the liver typically consumes 50–75 grams of fat to produce and secrete 100–150 grams of ketones per day. In effect, fat-derived metabolites replace carbohydrates as a fuel source. Typical mixed diets (non low-carbohydrate diets) are associated with a low level of blood ketones, typically less than 0.2 mM [12,13]. By contrast, nutritional ketosis starts at a blood level of beta-hydroxybutyrate (the predominant circulating ‘ketone’) of 0.5 mM and extends up to 5 mM [12,13]. Carbohydrate, and to a lesser extent protein, both inhibit liver production of ketones. The amount of carbohydrate that can be consumed while still promoting nutritional ketosis varies from person to person, but a general range is 20–50 grams per day, assuming protein is not consumed in excess. Thus, ketogenic diets are very low in carbohydrate and moderate/adequate in protein, translating into a carbohydrate level less than 50 grams per day and a protein level between 1.2 to 2.0 grams per kilogram of adjusted body weight. 

As defined by carbohydrate content, low-carbohydrate eating patterns could encompass approaches that vary widely in both total calories and protein and fat, which affects the percentage of macronutrients. Thus, it is preferred to define low-carbohydrate diets based on absolute amount of carbohydrate. 

## 3. Unintended Consequences of the DGA: The Obesity and Type 2 Diabetes Epidemic

Since the first DGA was released 40 years ago, there has been a consistent emphasis on limiting fat, especially saturated fat, and replacement of much of those calories with carbohydrate or polyunsaturated fat. Consequently, and over time, there has been an increase in the absolute intake of carbohydrate, resulting in a dietary pattern temporally associated with the marked rise in obesity, insulin resistance and type 2 diabetes (T2D) [3] as well as an increase in total mortality across multiple countries [14]. Today, more than two-thirds of American adults are overweight or obese [6], one-half have either prediabetes or T2D [7], and the numbers continue to rise. The economic burden of diabetes exceeds $300 billion per year [15]. Despite billions of dollars in investments by the private and public sectors, traditional drug and lifestyle treatments have had limited success in curtailing the obesity and diabetes epidemics. 

Indeed, excessive intake of carbohydrate was acknowledged and foreseen by previous Dietary Guidelines Advisory Committees (DGAC). The 2000 committee expressed concern that the government’s low-fat advice “could engender an overconsumption of total calories in the form of carbohydrates, resulting in the adverse metabolic consequences of high-carbohydrate diets,” adding, “Further, the possibility that overconsumption of carbohydrates may contribute to obesity cannot be ignored.” [16]. In 2015, the DGA Report explained that dietary advice should not emphasize reducing total fat, because low-fat/high carbohydrate “diets are generally associated with dyslipidemia (hypertriglyceridemia and low HDL-C concentrations” [17], which are indicators of increased risk for cardiovascular disease [18,19]. For this reason, the 2015 DGAC Vice Chair noted that “…there is no conventional message to recommend low-fat diets” [20]. However, despite removing the “low-fat” language from the 2015 and 2020 DGA, the current advice to consume between 20% and 35% of calories as fat is almost exactly the traditional low-fat diet, as commonly defined in the scientific literature [21]. 

One conclusion from this 4-decade long national experiment driven by the DGA is that the one-size-fits-all public health approach that encouraged people to eat less fat resulted in many Americans replacing fat calories with a greater amount of carbohydrate [3]. According to government data, since 1965, Americans have decreased fat intake by 25% and increased carbohydrates by 30%, expressed as percent of total energy [3]. Coupled with the fact that many Americans are insulin resistant, it is not surprising that only a small subset of the population has maintained metabolic health in the context of the current, de-facto low-fat dietary guidelines [22].

## 4. The Role of Carbohydrate in the Obesity and T2D Epidemics

Since fat is the most calorically dense macronutrient and excess body fat is the hallmark of obesity, low-fat and low-calorie diets have been the cornerstones of recommendations to manage both the obesity and T2D epidemics. By contrast, an alternative hypothesis is that the epidemics of obesity and T2D are driven by a systemic metabolic distortion of fuel partitioning as a result of overconsumption of sugars and starches, the two major categories of carbohydrate providing calories in the human diet. 

Metabolically, when dietary carbohydrate is replaced by fat, blood glucose and insulin do not increase as much after meals, facilitating a person’s metabolism to rely to a greater degree on fat for fuel. By contrast, carbohydrate intake is the most potent stimulant of the secretion of insulin, a lipogenic and anti-lipolytic hormone that promotes fat storage and strongly inhibits a person’s ability to mobilize and oxidize body fat. More specifically, insulin inhibits adipose tissue lipolysis and fatty acid oxidation – with effects that are both potent and immediate [23]. Over time, high carbohydrate consumption above a person’s tolerance overwhelms the body’s compensatory capacity to respond to persistent over-signaling from insulin, and, this, coupled with insulin’s role in the development of excess adiposity, can lead to a condition called insulin resistance. This further potentiates hyperinsulinemia, which is strongly linked to metabolic syndrome pathogenesis and a higher risk for cardiovascular disease [24]. 

In addition to the effects of chronic hyperinsulinemia on fat production and metabolic syndrome, the intake of added sugars containing fructose has also been shown to induce features of metabolic syndrome [25]. Fructose appears to be relatively powerful in its effects in this regard, likely related to its effect on energy levels in the liver and brain [26]. Experimental studies also suggest that a high glycemic response to carbohydrates may promote fat production by stimulation of insulin, and also by production of fructose via the polyol pathway, which then stimulates fat synthesis and accumulation [27].

These observations point to a role of high-carbohydrate intake, especially fructose, in the development of obesity, metabolic syndrome, and T2D. In support of this view, a burgeoning body of scientific evidence demonstrates that metabolic improvements are intimately connected with carbohydrate restriction [28,29]. Embracing this perspective that excessive carbohydrate intake is a fundamental driver of our obesity and T2D epidemics would represent a break from the DGA to date, yet it would allow for greater therapeutic flexibility, as people could personalize carbohydrate restriction each according to one’s metabolic needs. 

## 5. Obesity and T2D Are Conditions Strongly Associated with Insulin Resistance

Clinically, insulin resistance (IR) refers to a state in which a given concentration of insulin is associated with a suboptimal response [30]. Conditions highly associated with IR (e.g., metabolic syndrome, pre-diabetes, T2D) are identified by some combination of hyperglycemia and hyperinsulinemia. The molecular details are complex and diverse, but we know that most features can be triggered by the over-consumption of carbohydrate beyond the person’s capacity to use it for energy, and reversed by carbohydrate restriction [28,29], even before significant weight loss [31,32]. In this model, IR is correlated with, but not caused by, obesity. 

Insulin resistance is the primary feature underlying T2D that exists across a continuum in the general population. Insulin action in cells is disrupted to varying extents, which can cause a wide spectrum of signs and symptoms such as increased weight/adiposity, high blood pressure, high blood glucose, excessive circulating insulin, chronic inflammation, and dyslipidemia. A primary feature of IR is an impaired ability of muscle cells to take up circulating glucose, which manifests as persistently high blood glucose. The ability of insulin to suppress hepatic glucose production may also be impaired, further contributing to high blood glucose. Since the majority of dietary carbohydrate appears in the blood as glucose, it is apparent that individuals with IR have a fundamental problem metabolizing dietary carbohydrate. In response to an inadequate ability to clear glucose from the blood, a person with IR will divert a greater proportion of dietary carbohydrate to the liver, where much of it is converted to fat (i.e., *de novo* lipogenesis), as opposed to being oxidized for energy in skeletal muscle [33]. This greater conversion of dietary carbohydrate into fat, much of it entering the circulation as saturated fat [34], is an early metabolic abnormality that contributes to atherogenic dyslipidemia (i.e., high triglycerides, low HDL-C, and a predominance of small LDL particles), an atherogenic pattern that increases cardiovascular risk [18,19,35]. 

**Figure 1:**
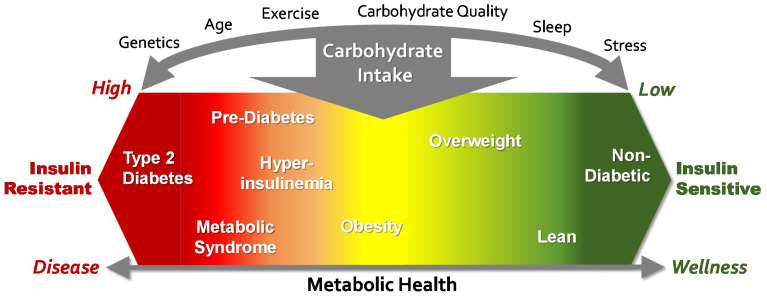
Expression of an insulin resistant or sensitive phenotype is a continuum that is strongly influenced by carbohydrate intake, with modulation based on genetic predisposition, age, and lifestyle choices.

As a general phenomenon, increasing carbohydrate intake is a driver that moves people toward an IR phenotype, whereas decreasing carbohydrate intake promotes metabolic health (Figure 1). In other words, the IR and insulin sensitive phenotypes are the opposite ends of a continuum whose expression is primarily driven by an increased or decreased carbohydrate intake, respectively. The thresholds of carbohydrate intake that move a person up or down this continuum of metabolic health may vary by genetic factors and may be modulated by age, lifestyle (e.g., exercise, carbohydrate quality, stress, sleep quantity/quality, etc.), and potentially gender, although there are few rigorous studies examining gender differences. Such a model fits with the growing body of evidence supporting low-carbohydrate diets as an effective tool to manage multiple metabolic impairments attributed to IR [28,29,31,32]. In fact, a very low-carbohydrate eating plan was the only successful therapy for T2D before insulin and other therapies became available [36]. Viewed through this lens, a large percentage of Americans may be metabolically positioned to benefit from a low-carbohydrate diet. Although individuals with IR may be expected to exhibit greater metabolic improvement, people across the insulin sensitivity spectrum respond favorably to a low-carbohydrate eating pattern [37,38,39,40].

Determining if a person is IR or carbohydrate intolerant and a good candidate for a low-carbohydrate or KD could be based on a number of observations and clinical tests. These measures can also be used to track progress over time. Standard clinical indicators of IR may include fasting glucose and insulin to calculate HOMA-IR, a glucose tolerance test including measures of insulin, or a diagnosis of prediabetes or type 2 diabetes based on fasting glucose or HbA1c. Other signs/symptoms of consuming carbohydrate at levels above an individual’s tolerance include weight gain (especially in the mid-section), dyslipidemia (high triglycerides, low HDL-C), poor success with low-fat diets, wide fluctuations in blood glucose after carbohydrate intake, and low energy levels during the day. 

## 6. Scientific Support for a Low-Carbohydrate Diet Option in the DGA

Low-carbohydrate diets have a long record of safe use. From a historical perspective, aboriginal hunting, fishing, and herding cultures survived for millennia with little available dietary carbohydrate [41,42,43]. A KD has been successfully used for 100 years in the treatment of epilepsy and diabetes [36,44,45,46], but this historical record of safe and therapeutic use has been overshadowed during the last half century by the introduction of pharmacologic management of these conditions as well as concerns regarding the intake of saturated fat at high levels. Quality long-term studies addressing safety and efficacy of very low-carbohydrate diets are lacking. However, aboriginal cultures such as the Inuit, Maasai, and Native Americans who had limited access to dietary carbohydrate maintained good health [41,42,43]. Two Arctic explorers who lived among the pre-contact Intuit in the Arctic were sequestered in a metabolic ward and then closely monitored as outpatients for a total of 12 months each [42]. Throughout this period, they ate a meticulously analyzed diet (15% protein, 80% fat, and <5% carbohydrate) patterned after that of the Inuit, and both maintained their health and function for the duration of the study. 

The metabolic and hormonal responses to a low-carbohydrate diet are associated with less oxidative stress and inflammatory responses after meals [12,47] as well as improvements in the features of IR and the metabolic syndrome [28,29,31,32]. These beneficial effects tend to increase in tandem with increased carbohydrate restriction. Evidence suggests that a KD may have unique therapeutic effects, owing in part to the increased endogenous production and availability of ketones which serve as both an alternative fuel and signaling molecule with wide-ranging health-promoting effects [48,49]. An increasing number of studies are now examining the basic science of ketones and their potential application across many indications (e.g., cancer, heart disease, neurological diseases, etc.). Ketones affect gene expression and pathways regulating inflammation, oxidative stress, immune function, membrane health, cell signaling, and antioxidant status [48,49,50]. 

Many different types of low-carbohydrate diets have been studied varying in total calories, the quantity and quality of carbohydrate, protein, and fat prescribed, as well as the level of education/support provided and adherence rates. For purposes of reviewing the published literature, the studies reviewed for this article share the common theme of aspiring to be carbohydrate restricted, generally targeting <130 grams of carbohydrate per day. Included among these studies are those intended to represent KD, which for most people require restricting carbohydrate to 30–50 g/day and, which may or may not have been verified by an objective measure of nutritional ketosis. As reviewed below, despite variability across studies in the formulation and implementation of diet interventions, a clear theme emerges — compared to low-fat diets, low-carbohydrate eating patterns result in equal or superior weight loss as well as the improvement of multiple established risk factors associated with IR and CVD [28,29,51]. Moreover, there may be unique, additional outcomes associated with KD including the superior benefits attributed to the increased availability of ketones that act both as a preferred fuel and a beneficial signaling molecule [48,49,50]. 

### 6.1. Obesity

While there is a body of literature examining the use of very low-calorie or semi-starvation KD (<800 kcal/day) in the medical treatment of obesity [11,52,53], the majority of more recent studies have involved mild caloric restriction. Several systematic reviews and meta-analyses have concluded that low-carbohydrate diets are at least as effective as low-fat diets for weight loss, and often more so [51,54,55,56,57,58]. Individuals who are insulin sensitive tend to respond well to either low-fat or low-carbohydrate diets, but those with insulin resistance tend to lose significantly more weight on the latter [59,60]. It is generally agreed that the primary driver of weight loss during a KD is greater satiety, resulting in a spontaneous reduction in calories [13,38,61,62,63]. Caloric restriction may be more sustainable on a low-carbohydrate diet because the lower insulin level and enhanced use of body fat for energy (including fatty acids and their derivatives, ketones) ensures increased mobilization of fat out of the fat tissues [23]. This results not only in weight loss but also more stable and efficient fuel delivery throughout the body, especially to the brain and the heart, and reduction in the wide excursions in blood glucose [64]. By contrast, low-fat diets usually require intentional caloric restriction as part of the dietary plan.

There is some initial water loss including reduced extra-vascular volume associated with the KD that contributes to rapid weight loss [65]. This loss of water is an expected positive outcome due primarily to the natural excretion of sodium (natriuresis) and fluid (diuresis) that occurs when insulin is reduced [66], which likely contributes to the blood pressure lowering effect of this eating pattern. Additional water is lost from metabolism of both intracellular glycogen (~3 grams of water is stored with each gram of glycogen) and fat, which can account for 2–3 kg weight loss during the first few weeks of a KD [65,67].

Whether weight loss is derived from fat-free mass or fat mass is important, as these have differing relationships to health [68]. Studies lasting beyond a few weeks that have measured body composition show a similar or greater loss of body fat in subjects on a low-carbohydrate diet compared to those on a low-fat diet [69,70,71,72]. Ketogenic diets also result in decreased visceral fat [38], which is highly associated with IR and metabolic impairment. In trials of very low-carbohydrate diets in adults with T2D, lean mass is preserved, and abdominal fat mass is reduced [73,74]. 

In the context of very low-calorie semi-starvation KD, a few studies have reported less protein sparing attributed to the KD [75,76], which could translate into a greater loss of lean mass over time. However, these studies [75,76] did not provide adequate protein and/or mineral replacement (sodium and potassium) [77]. Failure to compensate for the natriuretic effect of low-carbohydrate diets can lead to a general stress response (e.g., increased aldosterone, cortisol, catecholamine secretion), which may result in mineral imbalances (i.e., negative potassium balance) that adversely affect maintenance of lean tissue. A positive nitrogen balance on a KD, whether fed at a very low-calorie [78,79] or eucaloric [80] energy level, is achieved by ensuring adequate protein (i.e., ~1.2–2.0 g/kg ideal body weight) and minerals (see Section 7). 

This success of very low-carbohydrate eating patterns for achieving weight loss and favorable body composition stands in contrast to several large trials, funded by the National Institutes of Health, which demonstrated that weight loss on a low-fat diet is limited [81]. For perhaps this reason, the 2020 DGAC decided to exclude all studies on weight loss [82], despite widespread acknowledgment that weight reduction among overweight and particularly among obese individuals is crucial for both primary and secondary prevention of chronic disease [83]. Furthermore, the DGA itself has long held, as one of its three primary goals, the objective of helping Americans “reach and maintain a healthy weight,” [84] and the 2010 Dietary Guidelines stated, “[p]rimary prevention of obesity and related risk factors is the single most powerful public health approach to reversing America’s obesity epidemic over the long term.” [85]. 

Many long-term studies have shown no differences between low-carbohydrate and low-fat diet interventions after 1–2 years [86,87,88,89,90]. While it is tempting to conclude from these studies that the type of calories does not matter, these studies were associated with poor long-term dietary adherence and high attrition rates. Low-carbohydrate diet participants were allowed to increase their carbohydrate consumption as the trials progressed, making it likely that this reintroduction of carbohydrate blunted the benefits of carbohydrate restriction and led to weight regain. Despite similar weight loss among the comparison diets, the low-carbohydrate diets nevertheless consistently resulted in greater improvements in cardiometabolic risk markers [51,57]. 

### 6.2. Metabolic Syndrome

Metabolic syndrome is diagnosed when a person has at least three of the following physiologic signs: high triglycerides, low HDL-cholesterol, high fasting plasma glucose, high blood pressure, and high waist circumference [91]. Metabolic syndrome indicates a predisposition to T2D and cardiovascular disease. The condition has increased in parallel with higher carbohydrate intake over the last four decades, such that more than one in three American adults are now affected [92] and just one in eight Americans are metabolically healthy, where “healthy” is defined as having all five of these cardiometabolic risk markers in a normal range [22]. 

Dietary carbohydrate is a direct source of elevated blood glucose, which is the primary driver of insulin secretion. Therefore, low-carbohydrate diets naturally lead to fewer fluctuations in blood glucose and more stable insulin levels as evidenced in studies of individuals with T2D [74,93]. Consistent with the idea that a relative intolerance to carbohydrate is a common underlying feature of metabolic syndrome, clinical trials have shown that reductions in dietary carbohydrate, even without significant weight loss [31,32], result in improvements in the vast majority of cardiovascular and metabolic risk factors [28,29,51,56,57]. 

For example, outpatients with metabolic syndrome randomized to a 12-week KD lost more weight, total fat and abdominal fat compared to a matched group consuming a traditional low-fat, energy-restricted diet [12,13]. Patients consuming the KD also showed decreased serum triglycerides, increased HDL-C, decreased inflammatory markers and improved fatty acid composition profiles including lower circulating levels of saturated fat [12,13]. These experimental results point to the KD as a uniquely effective solution for addressing metabolic syndrome, with clear advantages over pharmaceutical approaches involving multiple drugs, often with significant cost and potentially harmful side effects [28,29]. 

### 6.3. Type 2 Diabetes (T2D)

A low-carbohydrate diet may provide exceptional benefits for T2D, which is essentially a disease of abnormal carbohydrate intolerance that affects more than 30 million Americans [7]. Even more alarming is the fact that 3-times that many people, or approximately 88 million U.S. adults, have prediabetes [7], that if left unchecked, can progress to T2D. Ketogenic diets were the treatment of choice for diabetes prior to the discovery of insulin in the early 1920s [36]. Insulin has been lifesaving for patients with type 1 diabetes. However, the use of insulin came at a high cost of weight gain as a side effect in patients with T2D, yet by the 1980s, this treatment, along with a low-fat, high-carbohydrate diet, had become the standard of care. Recently, the American Diabetes Association (ADA) has updated its nutrition recommendations to allow for more flexibility. Starting with their 2019 standards of care for patients with diabetes, the ADA stated that “Low-carbohydrate eating patterns, especially very low-carbohydrate (VLC) eating patterns, have been shown to reduce A1C and the need for antihyperglycemic medications. These eating patterns are among the most studied eating patterns for type 2 diabetes.” [94,95]

Low-carbohydrate and KDs have therefore re-emerged as a scientifically validated dietary pattern for individuals with T2D. In fact, there is good evidence supporting the use of low-carbohydrate diets as the first-line approach to treating T2D and as the most effective co-therapy with insulin in type 1 diabetes, partly because carbohydrate restriction decreases the requirement for insulin, and therefore the multiple adverse effects of insulin [10]. 

Individuals with prediabetes and T2D have greater intraday glycemic variability [96], which may exacerbate oxidative stress and vascular endothelial damage [97]. In people with T2D, glycemic variability has been tied to a higher risk of renal disease, macrovascular events, ulceration/gangrene, cardiovascular disease, and mortality [98]. Given the primary effect of carbohydrate on insulin secretion, it is not surprising that very low-carbohydrate diets are related to lower glycemic variability in people with type 1 diabetes [99,100] and T2D [64,74,93,101], which at a basic level enables many of the positive responses observed in clinical trials. 

Several well-controlled studies have evaluated the response of groups with T2D to low-carbohydrate and KDs over short- and long-term periods. After just 2-weeks of a low-carbohydrate diet in an inpatient setting, ten obese individuals with T2D demonstrated dramatic reductions in blood glucose and insulin levels, along with improved insulin sensitivity, and dyslipidemia [31]. Similar results have been reported over longer periods in outpatients [63,102,103,104,105,106]. For example, 363 pre-diabetic and diabetic subjects were offered either a standard low-fat/low-calorie diet or a KD for 6 months [106]. Weight loss and blood lipid changes were significantly better in the group receiving the KD. 

An even longer study on 262 adults with T2D who received telemedicine counseling on a KD by a health coach and physician-guided medication management team demonstrated that over half of the participants reversed their T2D after 1 year [104], where T2D reversal was defined as having a HbA1c below 6.5% while taking no diabetes medication or only metformin. Subjects also successfully reduced body weight, by an average of 12%, improved most of their cardiovascular risk factors, and 94% of subjects eliminated or reduced use of insulin medication [104,107]. The majority of participants in this trial have remained engaged in the program with patient retention of 83% at 1-year and 74% at 2-years [61]. In a similar longitudinal study using this telemedicine approach over 2-years, 96 patients with pre-diabetes experienced a 52% reversal of their pre-diabetes diagnoses [63]. 

Improvements in diabetes outcomes with KD are also associated with decreased healthcare costs. A large survey of adults following a low-carbohydrate eating pattern reported reductions in the need for medications related to glycemic control, hypertension, pain, depression, anxiety, and sleep, with 25% reporting lower medication costs [108]. In a retrospective examination of 67 insulin-dependent adults with T2D at one year, 40% were able to discontinue their long-acting insulin, and 88% were able to reduce their short-acting insulin These reductions were calculated to save more than $6,500 a year in insulin per patient [109]. In a 9000-patient primary care practice in the United Kingdom that prescribes the KD, the cost for glycemic-control medications was the lowest cost-per-patient among the other 19 medical practices in the area [110]. After 1-year in adults with T2D, glycemic control medications were reduced more in the very low-carbohydrate diet group compared to the moderate-carbohydrate or the usual care group [104,106]. 

These multiple trials from diverse groups have revealed that contrary to the conventional wisdom, T2D may not, in fact, be a chronic progressive disease. A T2D diagnosis can safely be reversed in many people using a very low-carbohydrate eating pattern, often while discontinuing insulin and other glucose-lowering medications. These findings were confirmed in a recent meta-analysis [111]. 

### 6.4. Cardiovascular Disease (CVD)

A substantial body of published work over the past 20 years has documented that low-carbohydrate diets induce favorable changes in cholesterol and other CVD risk markers, especially the cluster of abnormal risk factors associated with the IR phenotype, including high triglycerides, low HDL-cholesterol, increased small, dense LDL particles, high blood sugar, hyperinsulinemia, hypertension, and chronic inflammation [12,13,28,29,56,57,112]. 

For example, in a randomized, parallel trial comparing the effects of a low-carbohydrate diet to a low-fat diet in obese adults, the low-carbohydrate diet after 1-year resulted in greater weight and fat loss, a larger increase in HDL-cholesterol, and greater decreases in triglycerides and C-reactive protein as well as other markers of inflammation and endothelial dysfunction [113,114]. In another trial comparing a calorie-unrestricted low-carbohydrate diet to a reduced-calorie, low-fat diet in obese individuals with metabolic syndrome, the low-carbohydrate KD diet after 3-months resulted in a significant reduction in fasting and postprandial triglycerides, increased HDL-cholesterol, decreased small LDL particles, decreased glucose and insulin, improved vascular functioning as assessed by flow-mediated dilation of the brachial artery, decreased circulating saturated fatty acids, and lower concentrations of several pro-inflammatory meditators [12,64,115]. After 1-year, a group of participants with T2D following a KD showed a small increase in LDL-cholesterol (LDL-C), but robust improvement in the vast majority of CVD risk markers including decreases in triglycerides, small LDL particles, blood pressure, antihypertensive medications, C-reactive protein, white blood cell count, and the 10-year atherosclerotic cardiovascular risk score [107]. 

These examples are further supported by results from a meta-analysis concluding that low-carbohydrate diets significantly lowered the predicted risk of developing atherosclerotic CVD [57]. Although decreased body mass often accompanies low-carbohydrate diets, the broad-spectrum effects of low-carbohydrate diets on these CVD risk factors, including significant improvement in insulin sensitivity [31], are mostly independent of weight loss [31,32,40,115,116,117]. 

Chronic exposure to high levels of circulating insulin is a significant risk factor for CVD [118]. In non-diabetic adults, higher fasting and postprandial blood glucose and insulin are associated with substantially higher risk for CVD [119,120]. Reducing dietary carbohydrate, which is the primary driver of both blood glucose and insulin secretion, directly targets these problems. According to a meta-analysis that studied the relationship between insulin and CVD mortality in people without diabetes, those with the highest degree of IR compared to the lowest had a higher risk of CVD mortality [121]. Given the clear advantage of a low-carbohydrate diet in lowering circulating insulin throughout the day, these findings underscore the diet’s potential for reducing risk of CVD.

It is notable that LDL-C increases on average in response to a low-carbohydrate diet, although the effect is quite variable [122]. While the LDL-C concentration may either increase or decrease in different individuals depending on mostly unknown factors, a low-carbohydrate diet consistently shifts the LDL sub-fraction pattern to a less atherogenic profile, characterized by fewer small LDL particles [12,32,39,40,107,117,123,124]. This favorable shift happens even in the setting of high LDL-C concentrations as demonstrated in highly insulin sensitive elite athletes [37]. The isolated increase in LDL-C observed in some individuals consuming a low-carbohydrate diet needs to be understood in the broader context of improvements in multiple other well-established CVD risk factors. Furthermore, LDL-C, when lowered by a low-fat diet, has not been shown to have the same beneficial effect as lowering LDL-C with medications [125,126]. 

In the context of low-carbohydrate/high-fat diets, saturated fat is typically consumed in higher amounts, yet multiple studies have reported that circulating levels of saturated fatty acids stay the same or even decrease [12,13,32,124,127,128,129]. The primary reason for this phenomenon is that increased dietary saturated fat does not accumulate in the body even when intake is as much as 3-fold higher, due to the fact that metabolic adaptation to low-carbohydrate diets dramatically increases oxidation of these fatty acids [13,130] while at the same time decreasing hepatic production of saturated fatty acids from carbohydrate (i.e., *de novo* lipogenesis) [33,131]. 

Lower levels of circulating saturated fatty acids have relevance to CVD risk, because longitudinal studies consistently show that people with higher levels of circulating saturated fatty acids are at increased risk for developing metabolic syndrome [132], diabetes [133,134,135], heart failure [136], and mortality [137]. The observations that excessive circulating saturated fatty acids are a significant risk factor are consistent with in vitro and animal studies linking saturated fat to pro-inflammatory effects [138]. Saturated fat from dairy products, however, has no impact on metabolic or cardiovascular parameters in patients with T2D. In a recent randomized clinical trial, high-fat dairy food has similar impact on A1C, lipid profile, body weight and blood pressure in patients with T2D in comparison to low-fat dairy when total caloric consumption per day is equated [139]. 

High-carbohydrate, low-fat diets have been shown to be more likely to increase not only circulating saturated fatty acids but also the monounsaturated fatty acid palmitoleic acid (cis-16:1n7), which is also a product of *de novo* lipogenesis [34]. There is a remarkable stepwise uniformity in the response of circulating palmitoleic acid in response to varying carbohydrate intakes [13,32,124]. Likewise, palmitoleic acid consistently decreases when carbohydrates are restricted, especially on a KD [13,32,124]. Palmitoleic acid is therefore a useful proxy for the metabolic pathway that converts carbohydrate to fat. High palmitoleic acid in the blood or in tissue membranes is strongly linked to a host of metabolic derangements including obesity and metabolic syndrome [132,140], T2D [135,141,142], heart failure [136,143], and CVD mortality [137,144]. 

More than a billion people internationally have hypertension, and uncontrolled or untreated high blood pressure, which is the strongest risk factor for CVD and stroke [145]. Consistent with other markers of metabolic syndrome, a low-carbohydrate diet consistently decreases blood pressure in individuals with hypertension [58,104], which is likely mediated in part by lower circulating insulin levels and the associated natriuretic/diuretic effect described previously [66].

### 6.5. Low-Carbohydrate Diets and Mortality Outcomes

Concerns have been raised about the apparent association between low-carbohydrate diets and increased mortality. A search of Pubmed.gov yielded 14 such papers. Studies were excluded if there was no clear definition for “low-carbohydrate” [146,147,148] or if the paper did not isolate the link between a low-carbohydrate diet and health outcomes but instead reported on a score that combined intake measures of carbohydrate, fat and protein [149,150]. One systematic review was also identified, but that paper also did not report on the isolated link between a “low-carbohydrate” diet and health outcomes [151]. The remaining papers based their findings on cohorts from Japan [152], Sweden [153], the United Kingdom [154], and the United States [155,156,157,158,159,160]. In these 9 papers, “low-carbohydrate,” as a percent of total energy, is defined as follows (listed in order of the citations in the previous sentence): 53% (Japan), 40% (Sweden), 40.9% (United Kingdom). 37%, 39%, 47.3%, 40%, 37.2%, and 43.2% (United States). None of these numbers falls within the current definition of a low-carbohydrate diet, which allows for carbohydrates at 30% of energy or less. Thus, these studies cannot be characterized as representing a true low-carbohydrate diet, and their conclusions cannot be viewed as relevant to the low-carbohydrate scientific literature. Interestingly, in the largest study published to date which included 135,335 individuals across 18 countries, higher carbohydrate intake was associated with an increased risk of total mortality, although this study too was not designed to test low-carbohydrate diets [14].

### 6.6. Qualitative Research

Qualitative and survey research has shown that adults consuming a low-carbohydrate eating pattern have positive health outcomes such as less hunger, greater energy, and improved health, but that lack of support from family and physicians can be a barrier to adherence [161,162]. Qualitative surveys of healthcare providers reveals that many practitioners have found low-carbohydrate diets to be helpful for their patients and as a consequence, have changed the way they view and practice healthcare [163].

In summary, an increasing body of scientific evidence indicate that low-carbohydrate diets are uniquely effective for combating IR, a root cause of obesity, metabolic syndrome, prediabetes, and T2D that affects well over 100 million Americans [6,7]. 

## 7. Principles of Very Low-Carbohydrate (Ketogenic) Diets

There are many different types of low-carbohydrate eating patterns that can vary in the quantity and quality of macronutrients. In general, a greater degree of IR and carbohydrate intolerance requires a greater level of carbohydrate restriction to manage this condition effectively, but the quality as well as the quantity of carbohydrate are both important considerations. Effective management of IR and its multiple manifestations may be improved by substituting lower quality carbohydrates with higher quality ones. For example, limiting simple and added sugars, especially fructose, as well as high-glycemic, overly processed, nutrient-depleted carbohydrate sources in favor of lower glycemic, nutrient-rich, whole foods (e.g., non-starchy vegetables, legumes) is likely to yield benefits on IR. The glycemic index is a method of determining the quality of carbohydrate-containing foods based on the 2-hr postprandial blood glucose response. High-glycemic foods raise blood glucose to a greater extent than low glycemic index food. The many variations and nuances of diets containing different amounts and sources of carbohydrate-containing foods are complex and beyond the scope of this review. However, since educational content specific to low-carbohydrate diets is absent from nearly all training of healthcare professionals, including dietitians, we provide a general overview of important considerations in designing the most carbohydrate-restricted subset of low-carbohydrate eating patterns (i.e., a KD) aimed at achieving nutritional ketosis.

The formulation of safe, effective, palatable, and sustainable KD entails relatively simple adjustments in conventional diets, focused primarily on replacing sugar- and carbohydrate-dense foods with un-processed, low-carbohydrate/high-fat foods. Proper formulation of a KD entails restriction of carbohydrate and intake of adequate—but not high—protein and sufficient minerals to offset the natriuretic effect of ketosis and lower insulin levels. Counting calories is usually not necessary. Several studies demonstrate that obese individuals in nutritional ketosis instructed to eat to satiety, with no specific caloric prescription, spontaneously eat less and achieve sustainable weight loss [13,38,61,62,63]. 

## 8. Macronutrients

### 8.1. Carbohydrate

Carbohydrate, and to a far lesser extent, protein are the two primary dietary factors stimulating blood glucose and insulin responses while inhibiting blood ketones. The amount of time and level of carbohydrate restriction that are needed to normalize blood sugar and achieve nutritional ketosis vary widely from person to person. Nutritional ketosis usually requires less than 50 grams per day of carbohydrate but may range from 30 to >70 g/day across individuals [38]. Generally, the more overweight or IR the person at the start of the diet, the greater degree of carbohydrate restriction is needed to normalize blood glucose and insulin. The time needed for the body to achieve full metabolic adaptation to a KD takes at least several weeks if not months [130].

A wide range of nutrient-rich whole foods can be incorporated into KD, including non-starchy vegetables, meats (beef, chicken, pork, fish, shellfish, lamb), nuts and seeds, fruit oils (olive, avocado, coconut), cheeses, butter, cream, whole eggs, and small amounts of fruits (berries, olives, avocado, tomatoes, lemons/limes). Depending on the individual and the degree of carbohydrate restriction, the approximate daily carbohydrate allotment in terms of food sources generally breaks down as follows on a KD:

- 5–10 g from protein-based foods. Eggs, cheese, and shellfish will carry a few residual grams of carbohydrate from natural sources and added marinades and spices.
- 10–15 g from non-starchy vegetables.
- 5–10 g from nuts/seeds. Most nuts contain 5–6 g carb per ounce.
- 5–10 g from fruits such as berries, olives, tomatoes, avocados.
- 5–10 g from miscellaneous sources such as low-carb desserts, high-fat dressings or drinks with very small amounts of sugar.

### 8.2. Protein

Consuming too much protein will prevent a person from achieving nutritional ketosis, while consuming too little protein will adversely affect meal acceptability/satiety and potentially lead to loss of muscle mass and function. Target protein intakes are typically between 1.2 and 1.5 g/kg body weight. There is little evidence to support protein intakes higher than 2.0 g/kg, and such high levels of protein will make it harder to achieve nutritional ketosis. In the context of a weight maintenance KD, this level of protein is approximately 15–20% of the individuals’ daily energy expenditure, which is similar to the current average protein intake in the standard American diet. 

In people with excess adiposity, using actual body weight is likely to result in protein being over-prescribed. In these cases, use of ideal body weight (IBW) or adjusted body weight is warranted [164,165], although correction formulas are also limited in accuracy due to individual variations in physical activity, muscle mass, health status, and other factors influencing protein metabolism and requirements. The World Health Organization recommends a healthy body mass index (BMI) of 18.5 – 25 g/km^2^, which can be used to determine an IBW range for any given height. Adjusted body weight = IBW + [(current weight – IBW) × 0.25]. 

Those who engage in moderate or vigorous activity may benefit from a slight increase in protein consumption, but the recommended range noted above is more than adequate to meet the needs of most active individuals with goals of muscle gain. Individuals with T2D typically lose 8% of their lean muscle mass every decade from age 40 and 15% per decade from age 70 [166]. Thus, ensuring adequate protein intake is important to offset this loss of muscle. Resistance training may be considered as a form of activity that helps preserve and build lean tissue, even in older adults [167]. 

Based upon published cardio-metabolic health responses to a well-formulated KD, there is no objective evidence in favor of avoiding animal protein consumed in moderation. However, individuals who choose a lacto-ovo vegetarian or even a vegan low-carbohydrate diet can do so successfully. If eggs and dairy proteins are restricted, attention to quality protein sources to achieve adequate essential amino acid intakes is warranted. And, as is true for any vegetarian diet that excludes eggs and fish, dietary supplements may be necessary to cover vitamin B12 and long-chain omega-3 fatty acid requirements. 

### 8.3. Fat

Determining the appropriate amount of fat to eat on a KD is best achieved by encouraging people to eat to satiety. Emphasis should be placed on foods high in monounsaturated and saturated fatty acids while limiting sources rich in omega-6 polyunsaturated fatty acids (e.g., seed oils such as soybean, peanut, safflower, sunflower, corn). The primary functions of dietary fat in the context of a KD is to serve as fuel, add flavor and pleasure to meals, and to promote satiety. While omega-6 polyunsaturated fats are essential, the amount needed to meet this requirement is very small. Empirically, concentrated sources of polyunsaturated fats are not well tolerated at the high levels of fat consumed on KD due to gastro-intestinal symptoms. By contrast, monounsaturated and saturated fats are optimal fuels and should comprise most of the fat consumed. As noted previously, while KD that are higher in saturated fat can lead to increased circulating LDL-C, there is a net benefit on CVD risk factors in at-risk individuals (e.g., T2D) [28,29,51,56,57]. A minority of individuals, however, experience a marked increase in LDL-C, and it remains unknown whether this poses any long-term risk or if these individuals should limit foods with high saturated fatty acid content (e.g, fatty meats, full fat dairy products). These “hyper-responders” usually experience other clinical benefits attributed to the KD (e.g., weight loss and improvement in markers associated with IR). Finally, maintaining a good source of the long-chain omega-3 fatty acids, eicosapentaenoic acid and docosahexaenoic acid, is also important. This can be achieved by consuming fatty fish (salmon, tuna, sardines, etc.) twice per week. 

## 9. Micronutrients

A well-formulated low-carbohydrate/KD is not associated with micronutrient deficiencies [168]. When composed of unprocessed, natural foods, this diet contains adequate essential vitamins and minerals achieved through the consumption of a wide variety of whole foods prepared using appropriate methods to preserve nutrients. Certain medical conditions or avoidance of specific foods on a KD may necessitate supplementation. Below is a discussion of some of the most relevant nutrients that may require special attention. 

### 9.1. Sodium and Potassium

Ensuring adequate sodium intake is particularly important because lower insulin and nutritional ketosis trigger increased excretion of sodium along with fluids. This natriuretic effect leads to the loss of both sodium and fluid, which, if not replaced, can have side effects. For many people who achieve nutritional ketosis, losing extra fluids results in perceived benefits such as rapid weight loss, reduced/eliminated need for diuretic medication, lessening of edema, and improved blood pressure. However, once the excess fluid has been cleared, this natriuretic effect of nutritional ketosis persists, causing continued losses of sodium and reduced blood plasma volume. Consequences can include dizziness, orthostatic hypotension, fainting, fatigue, constipation, and headaches. Other potential consequences are adrenal stress, characterized by increased aldosterone, cortisol, and epinephrine. Aldosterone acts on the kidneys to increase sodium reabsorption to restore sodium balance, but in so doing accelerates the loss of potassium. Thus, sodium restriction on a KD can lead to potassium wasting by the kidneys. Negative potassium balance manifests as muscle twitches, cramps, irregular heartbeats, neuromuscular dysfunction, and loss of muscle mass. 

Countering these potential side effects simply requires consuming adequate sodium and potassium. An additional 1–2 grams of sodium is generally needed beyond the normal consumption of about 3 g/day, for a total of 4–5 g/day for non-hypertensive individuals [169]. Recent research also indicates that an optimal target for potassium intake for adults is 4 grams per day [169]. The best sources of potassium are vegetables and homemade broths. Other good sources are avocados, nuts/seeds, canned salmon, and unprocessed meats. Intra-cellular potassium is released during cooking, so it is important not to discard nutrient-rich drippings when preparing meats, and to steam rather than boil vegetables. Thus, adequate sodium and potassium intake, which can be achieved though the selection of appropriate food sources and cooking methods, along with careful monitoring of symptoms, is critical to avoid potential side effects and optimize a person’s ability to enjoy and continue with a ketogenic eating plan.

While ensuring adequate sodium intake is important, there is also increasing evidence that high salt intake may increase the risk for obesity, hypertension, and metabolic syndrome [170]. The mechanism appears to be that rising serum osmolality triggers the production of fructose [170,171]. These negative effects of high sodium intake can be reversed by hydration [171,172]. A recommendation of six to eight glasses of water a day, in addition to other fluid intake, is recommended.

### 9.2. Calcium

The recommended dietary allowance for calcium in adults is 1000–1200 mg/day. The primary and best source of calcium is dairy foods. Since many dairy foods like milk and yogurt contain several grams of carbohydrate, the best source of calcium on the KD is cheese, especially hard cheeses, such as parmesan, cheddar, gouda, and provolone, which contain virtually no carbohydrate. Green vegetables like broccoli, spinach, and kale also have calcium but less so than cheese and in a less bioavailable form. Other sources of calcium on a ketogenic diet include sour cream, tofu, sardines with bones, nuts/seeds, and home-made broths made from chicken or beef, including the bones. A calcium supplement is generally not needed on this diet if foods with calcium are consumed, but a supplement may be considered for people at risk for osteoporosis. 

### 9.3. Magnesium

Magnesium is an essential mineral. Because it is often lost during food processing, marginal deficiency of this nutrient is not uncommon in the general population. Diuretic medications and heavy use of alcohol also deplete magnesium. Magnesium has a key role in muscle and nerve transmission. Since most magnesium is contained within cells, serum tests for magnesium are of little value. Deficiency can result in muscle twitching and spasms or cramps, as well as persistently low blood potassium levels. Good sources of magnesium include dark green vegetables, nuts/seeds, non-processed meats, and homemade broths. It is important to capture the drippings from meat to retain magnesium. Magnesium depletion is common in individuals with T2D, in part due to increased urinary excretion [173]. Because magnesium depletion impairs glucose control [174], it is often necessary to provide supplemental oral magnesium in combination with KD in order to optimize T2D reversal. 

### 9.4. Vitamin D

It is increasingly apparent that many people are marginally deficient in vitamin D based on serum levels of 25-hydroxyvitamin D [175]. This may reflect less sun exposure and use of sunscreens, which limit the natural vitamin D synthesis that occurs with sun exposure. Vitamin D fortified milk is not recommended in appreciable amounts on KD due to its 50 grams per liter of sugar content. Food sources of vitamin D include fatty fish such as salmon, egg yolks, and cheese. For people who do not get regular sun exposure, a vitamin D supplement or use of a multivitamin that includes vitamin D (~1000 IU) may be necessary to bring serum levels into an acceptable range. 

### 9.5. Fiber

The beneficial effects of fiber are attributed mainly to its ability to slow absorption of glucose, promote satiety, and contribute to the bacterial production of short-chain fatty acids, principally butyrate. Butyrate is a preferred energy source of intestinal cells and is associated with well-documented effects on gut health. However, the need for ample fiber on a KD is less clear, since the diet inherently decreases postprandial glucose and insulin while promoting satiety. Low fiber intake would likely result in decreased bacterially produced butyrate, but KD accelerate endogenous production of beta-hydroxybutyrate in the liver, estimated to be in the range of 100–150 grams per day during nutritional ketosis [176]. Ketones are short-chain fatty acids that can function like butyrate as a preferred energy source and a signaling molecule to promote gut health [177]. From this perspective, nutritional ketosis may promote gut health. It should be noted that KD are not devoid of fiber. Inclusion of non-starchy vegetables and 1–2 ounces of nuts/seeds results in ~15–20 grams of fiber per day, which appears to be sufficient. Controlled studies of fiber in the context of a KD have not yet been conducted.

## 10. Summary

Many Americans have varying degrees of IR as evidenced by the high prevalence of obesity, metabolic syndrome, prediabetes, and T2D, which have all been demonstrated in a large body of scientific literature to be highly responsive to a low-carbohydrate eating pattern. A broad range of markers linked with the IR phenotype and associated with an increased risk of CVD are also improved by a low-carbohydrate approach. The 2020 DGAC stated that its review process did not find any studies of KD and only one study of low-carbohydrate diets. It appears that unrealistic inclusion criteria for the literature search resulted in the dismissal of a large and credible body of published research. Furthermore, while the stated purpose of the 2020 DGA is to provide dietary advice for "healthy" Americans, the high proportion of Americans with IR makes the case for redefining the target population of the guidelines to include this majority of Americas who would likely benefit from the inclusion of a low-carbohydrate dietary option. 

## Data Availability

No original data were generated for this manuscript.

## Abbreviations

Acceptable Macronutrient Distribution Ranges (AMDR), American Diabetes Association (ADA), cardiovascular disease (CVD), Dietary Guidelines for Americans (DGA), Dietary Guidelines Advisory Committees (DGAC), high-density lipoprotein cholesterol (HDL-C), ideal body weight (IBW), insulin resistance (IR), ketogenic diet (KD), low-density lipoprotein cholesterol (LDL-C), type 2 diabetes (T2D), very low-carbohydrate (VLC).
